# X-ray structure of engineered human Aortic Preferentially Expressed Protein-1 (APEG-1)

**DOI:** 10.1186/1472-6807-5-21

**Published:** 2005-12-14

**Authors:** Babu A Manjasetty, Frank H Niesen, Christoph Scheich, Yvette Roske, Frank Goetz, Joachim Behlke, Volker Sievert, Udo Heinemann, Konrad Büssow

**Affiliations:** 1 Protein Structure Factory, c/o BESSY GmbH, Albert-Einstein-Str. 15, 12489 Berlin, Germany; 2 Max-Delbrück-Centrum für Molekulare Medizin, Robert-Rössle-Str. 10, 13092 Berlin, Germany; 3 Institut für Chemie/Kristallographie, Freie Universität, Takustr. 6, 14195 Berlin, Germany; 4 Protein Structure Factory, Heubnerweg 6, 14059 Berlin, Germany; 5 Max-Planck-Institut für Molekulare Genetik, Ihnestr. 73, 14195 Berlin, Germany; 6 Charité Universitätsmedizin Berlin, Institut für Medizinische Physik & Biophysik, Ziegelstr. 5-9, 10098 Berlin, Germany; 7 Structural Genomics Consortium, University of Oxford, Botnar Research Centre, Oxford, OX3 7LD, UK; 8 Case Centre for Proteomics, Case Western Reserve University, Upton, New York 11973, USA

## Abstract

**Background:**

Human Aortic Preferentially Expressed Protein-1 (APEG-1) is a novel specific smooth muscle differentiation marker thought to play a role in the growth and differentiation of arterial smooth muscle cells (SMCs).

**Results:**

Good quality crystals that were suitable for X-ray crystallographic studies were obtained following the truncation of the 14 N-terminal amino acids of APEG-1, a region predicted to be disordered. The truncated protein (termed ΔAPEG-1) consists of a single immunoglobulin (Ig) like domain which includes an Arg-Gly-Asp (RGD) adhesion recognition motif. The RGD motif is crucial for the interaction of extracellular proteins and plays a role in cell adhesion. The X-ray structure of ΔAPEG-1 was determined and was refined to sub-atomic resolution (0.96 Å). This is the best resolution for an immunoglobulin domain structure so far. The structure adopts a Greek-key β-sandwich fold and belongs to the I (intermediate) set of the immunoglobulin superfamily. The residues lying between the β-sheets form a hydrophobic core. The RGD motif folds into a 3_10 _helix that is involved in the formation of a homodimer in the crystal which is mainly stabilized by salt bridges. Analytical ultracentrifugation studies revealed a moderate dissociation constant of 20 μM at physiological ionic strength, suggesting that APEG-1 dimerisation is only transient in the cell. The binding constant is strongly dependent on ionic strength.

**Conclusion:**

Our data suggests that the RGD motif might play a role not only in the adhesion of extracellular proteins but also in intracellular protein-protein interactions. However, it remains to be established whether the rather weak dimerisation of APEG-1 involving this motif is physiogically relevant.

## Background

Arterial smooth muscle cells (SMC) are essential for the formation and function of the cardiovascular system. Abnormalities in their growth can cause a wide range of human disorders such as atherosclerosis, the principal cause for heart failure, thus the leading cause for deaths in the western world [1-3]. The molecular mechanisms that regulate SMC growth and differentiation are unclear partly due to the lack of specific markers and defined *in vitro *differentiation systems [4]. The recently discovered Aortic Preferentially Expressed Protein-1 (APEG-1) may serve as a sensitive marker for vascular SMC differentiation. APEG-1 is expressed in differentiated vascular SMC *in vivo *and was found to be down-regulated rapidly in de-differentiated vascular SMC *in vitro *and in injured arteries *in vivo *[5,6].

Recently, three additional, larger products of the APEG-1 gene have been identified in rodents: in striated muscle, SPEGα and SPEGβ, and in the brain, BPEG [7]. The originally discovered APEG-1 mRNA is transcribed from a different promoter than the SPEGβ mRNA. This promoter is located between two exons of the much larger SPEGβ open reading frame. SPEGβ contains a serine/threonine kinase domain, and several immunoglobulin and fibronectin structural domains. The immunoglobulin sequences and the pattern of surrounding domains of SPEG proteins have significant homology with the smooth muscle myosin light chain kinase (smMLCK) and the giant muscle protein titin. Therefore, it has been hypothesized that all four protein products of the APEG-1 gene (APEG-1, BPEG, SPEGα and SPEGβ) are part of the functionally and structurally diverse smMLCK protein family [7].

**Figure 1:**
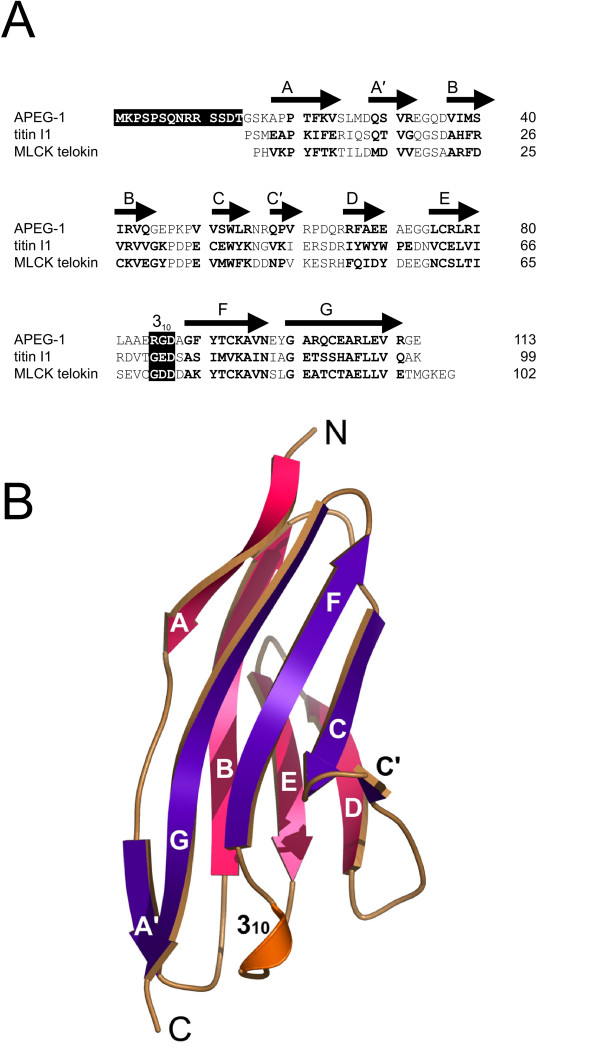
**Structure and sequence alignment of APEG-1**. **A**: Alignment of APEG-1 with the I1 domain of titin (PDB 1G1C) and the telokin domain of MLCK (PDB 1FHG). The β-strands are labeled according to Ig fold I set nomenclature. The N-terminal 14 residues and the adhesion recognition RGD motif are highlighted. **B**: Ribbon diagram of the ΔAPEG-1 monomer. The front sheet (strands A'GFCC') and back sheet (strands ABED), are colored purple and pink, respectively. The 3_10 _helix is shown in orange.

The amino acid sequence of APEG-1 (SwissProt Q15772) defines a single Ig-like domain (Figure 1A). Ig-like domains adopt a Greek-key β-sandwich fold and contain two β-sheets that pack against each other. In Ig-like domains of the I-set, one sheet is composed of four β-strands (ABED) and the other comprises five β-strands (A'GFCC') [8]. A disulfide bond is formed between strands B and F in most of the extracellular Ig domains which is essential for their structural integrity [9] whereas intracellular Ig domains are stabilized by a hydrophobic core [10,11]. Biochemical studies suggest that APEG-1 is a nuclear protein [5] despite the as yet unrecognized nuclear localization signal [12]. Ig domains interact with a wide variety of other proteins either by end-to-end contacts of the loops from opposite ends of the β-sandwich or by sheet-sheet contacts [13].

A PROSITE database [14] search revealed that APEG-1 contains an Arg-Gly-Asp (RGD) adhesion recognition motif. The RGD motif is found in a number of proteins that play a role in cell adhesion, including some forms of collagens, fibrinogen, vitronectin, von Willebrand factor (VWF), snake disintegrins and slime mold dicoidins (PROSITE: PDOC00016). The RGD sequence is also found in several important extracellular matrix proteins and serves as an adhesion ligand for members of the integrin family of cell-surface receptors [15-17]. Experimentally determined structures of cell-adhesion proteins reveal that the RGD motif is localized within loop regions and can adopt a broad set of conformations [18].

The Protein Structure Factory [19] is developing novel strategies to address targets of its *Homo sapiens *structural genomics effort which initially failed to yield good quality crystals. In this paper, we describe the success in crystallization of APEG-1 by truncation of an amino-terminal region that is predicted to be disordered according to bioinformatic analyses [20,21].

## Results

### Protein production strategy

Since the full-length APEG-1 could not be crystallized, a novel strategy for protein production [21,22] was applied. Structurally disordered regions of the protein were predicted with COILS, REMARK465 and HOT LOOPS from the DisEMBL™ package [23] and with PONDR^® ^[24]. The program PONDR^® ^predicted disorder at the N-terminus up to amino acid 18 whereas COILS and REMARK465 predict a disordered stretch up to amino acid 23. However, due to the start of the Ig domain at residue 20 (SwissProt Q15772, Figure 1A) disorder is unlikely beyond this point.

PONDR^® ^predicted thirteen C-terminal residues to be disordered which is contradictory to the results obtained with COILS which predicts order in this region. COILS has been proposed to be effective as a filter to remove false positive predictions made by other networks [23]. These analyses show that it may be helpful to consider more than one disorder prediction algorithm for correct assignment. In the structure, the C-terminus was later found to be ordered except for the very last amino acid.

The N-terminus was truncated by 14 residues, and the truncated (ΔAPEG-1) protein was prepared which showed monodispersity like the full-length protein. Similar thermal stability was determined by differential scanning calorimetry for both proteins; the midpoints of the unfolding transition were 56.3°C and 54.4°C for ΔAPEG-1 and APEG-1, respectively.

### Structure description and comparison

The truncation approach proved successful, and the crystal structure of ΔAPEG-1 was determined to a resolution of 0.96 Å (Figure 1B).

We searched public databases for high resolution structures of immunoglobulin domains. The immunoglobulin structure with the highest resolution as yet was found to be PDB 1K5N (1.09 Å) [25].

**Figure 2:**
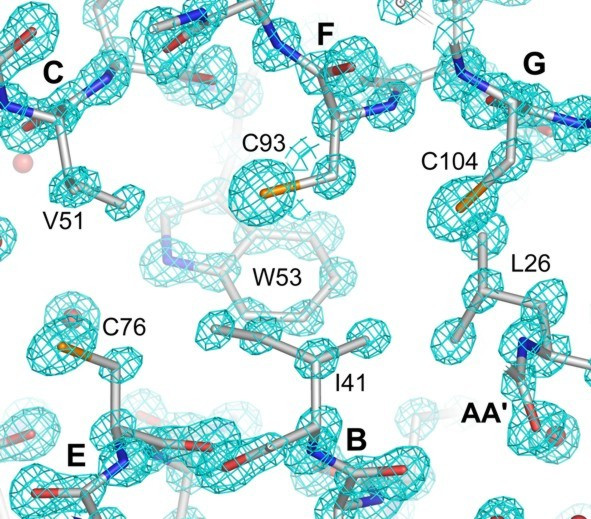
**Cross section of the hydrophobic core**. Electron density map contoured at 3σ.

The structure of APEG-1 shows the intermediate set (I set) immunoglobulin superfamily fold [13] which consists of a β-sandwich forming a central hydrophobic core. The front sheet comprises strands A'GFCC' which are arranged with A' and G parallel and GFCC' anti-parallel, whereas the back sheet comprises strands ABED oriented anti-parallel to one another. In addition, one 3_10 _helix containing the RGD sequence lies near the C-terminus. The main hydrophobic core of the domain is formed around the aromatic residues W53, F22 and Y91 (Figure 2).

The structural topology of ΔAPEG-1 closely resembles that of the prototypical I set domains of the Ig fold superfamily [26], such as those found in telokin (PDB 1FHG, 1TLK; r.m.s.d = 0.9 Å; 33.3% sequence identity) [27] and the I1 domain of the striated muscle protein titin (PDB 1G1C; r.m.s.d = 1.1 Å; 26% identity) [28] (Figure 1A). Telokin is identical to the C-terminal domain of myosin light chain kinase (MLCK) which is a key enzyme in the regulation of contractile activity in smooth muscle. Both APEG-1 and telokin are proteins that contain an Ig-like domain and represent a small portion of a larger muscle kinase (SPEGβ and MLCK, respectively) [7].

A disulfide linkage is normally present in extracellular immunoglobulin domains between strands B and F. In ΔAPEG-1 this is replaced by hydrophobic interactions between residues I41 and C93 in strands B and F, respectively (Figure 2). The other main hydrophobic residue pairs anchored in the core are V51 (strand C) – C76 (strand E), C104 (strand G) – S25 and L26 (loop between strands AA'), and M39 (strand B) – Y91 (strand F). The superposition of the structures of telokin, the titin I1 domain and ΔAPEG-1 reveals that the residues which compose the hydrophobic core are highly conserved in arrangement and packing volume.

Telokin contains four cysteines in the core which have the potential to form two disulfide bonds. Three of these cysteines are also present in ΔAPEG-1 (Figure 1A). A disulfide bridge is observed in the structure of the titin I1 domain which was described as the first disulfide bridge found in an intracellular Ig domain [28]. This bridge connects β-strands C and E (C37-C62) and thus the front and back sheets of the I1 β-sandwich. Only one cysteine of this bridge is present in ΔAPEG-1 and no disulfide linkages were found (Figure 2). The alternative side chain conformation of C93 bears the potential to form a disulfide bond with C104 which would link the adjacent strands F and G of the front sheet of the β-sandwich. However, modeling with Coot [29] resulted in an S-S distance of 2.6 Å, which is considerably longer than the normal disulfide bond of 2.0–2.1 Å.

The residues forming the hydrogen bonds and salt bridges at the interface region – E33, R55, R65 and D87 of the RGD motif – are highly conserved in the three proteins. However, a similar homodimeric arrangement was not observed in the structure of the I1 domain or telokin.

### Homophilic interaction

**Figure 3:**
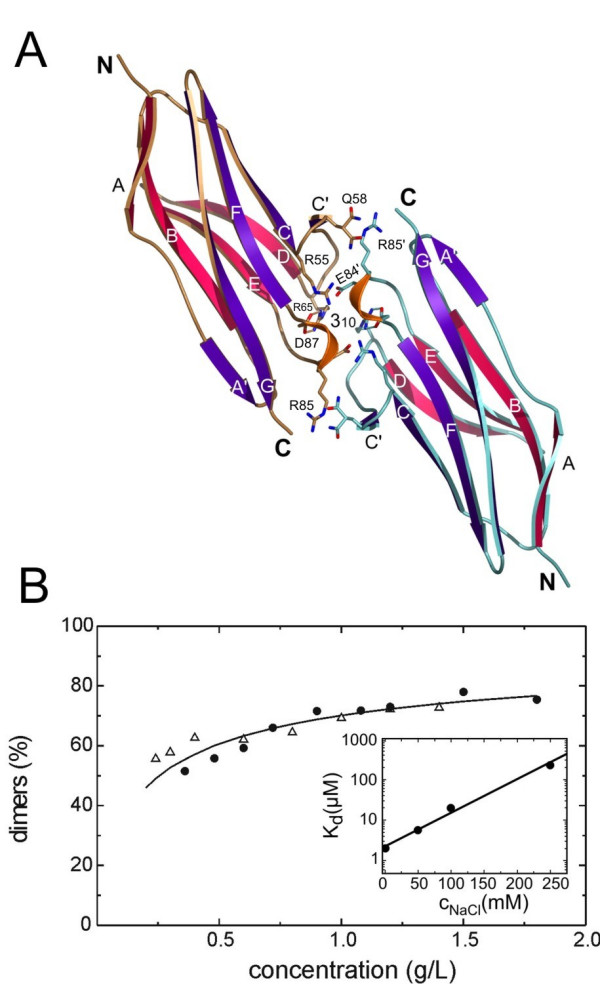
**Quaternary structure of APEG-1 and ΔAPEG-1**. **A**: Ribbon diagram of the ΔAPEG-1 homodimer. Bi-dentate salt bridges are represented by broken lines. Residues lined up at the binding interface are depicted. **B**: Analytical sedimentation equilibrium ultracentrifugation plot of determined molecular mass versus concentration of APEG-1 (white triangles) and ΔAPEG-1 (black spheres) at 100 mM NaCl. The mass of the respective homodimer is set to 100%. **Inset**: Dissociation constants of ΔAPEG-1 at different NaCl concentrations.

ΔAPEG-1 crystallized as a monomer in the asymmetric unit and an accessible surface area (asa) of 5775 Å^2 ^was found for a single subunit. Each ΔAPEG-1 molecule forms three distinct inter-protein contacts with neighbors, burying surface areas of 11.2% (648 Å^2^), 7.8% (420 Å^2^) and 6.7% (387 Å^2^). These values are all within the range of buried asa observed in protein dimer structures (6.5–29.4%) [30]. The large contact regions suggest that ΔAPEG-1 can form homophilic interactions. The homodimer with the largest buried surface area utilizes end-to-end packing with the subunits' N-termini pointing in opposite directions (Figure 3A). The residues involved in formation of this dimer are exclusively located within the loop cluster between strands CC', C'D and EF which lie close to the C-terminus and within the 3_10 _helix which contains the RGD motif. These loops are interconnected by salt bridges and a number of hydrogen bonds. The dimer interface includes two buried bi-dentate salt bridges – involving the RGD motif – between R65-D87 and R65'-D87' (the apostrophe denotes the adjacent ΔAPEG-1 molecule). These buried salt bridges are further stabilized by salt bridge interactions between E84-R65' and E84'-R65, respectively, and the interactions between E33-R66' and R66-E33'. Additionally, the dimer interface has several van der Waals interactions and water-mediated hydrogen bonds between residues R85, G86, R55, Q58 and the symmetry-related mates R85', G86', R55', Q58' which further stabilize the dimer interface. The backbone oxygen atom O of G86 is bonded to N^ζ2 ^of R55, the last residue of strand C, and the backbone amide of R85 to the oxygen O^ε2 ^of E33. A strong, but unfavorable, interaction between E84-D63', E84'-D63 is also observed at the dimer interface.

The quaternary structure was investigated by sedimentation equilibrium in an analytical ultracentrifuge. An average dimer dissociation constant of *K*_d _= 20 μM derived from the concentration-dependent *M*_w _measurements was obtained for both full-length APEG-1 and ΔAPEG-1 at 100 mM NaCl, demonstrating the nearly identical amount of dimers (Figure 3B). The dissociation constant was determined as a function of the salt concentration, and a pronounced correlation was found, as expected for interactions which are stabilized by salt bridges (Figure 3B, inset). The dissociation constant is increased hundred fold – from 2 μM to 200 μM – when the salt concentration is increased from 0 to 250 mM.

## Discussion

Prediction and removal of disordered regions proved to be a successful strategy for the crystallization and structural analysis of APEG-1. We assume that the flexible, unstructured N-terminus was the reason for earlier failures to crystallize the full-length protein. The I set of Ig proteins is characterized by a hydrophobic core that is important for their stability and activity [11]. The examination of the hydrophobic cores of several I-set proteins reveals that they are flexible and can tolerate considerable variation of hydrophobic residues particularly on the edges of the core [8,31]. The absolute invariants within the core are tryptophan (W53) and tyrosine (Y91). A comparison of the structure with I-set domains of the muscle proteins telokin and titin reveals that residues get shuffled within the β-sheets causing local conformational changes in the side chains while the closely packed hydrophobic core is maintained. A disulfide bond links the two β-sheets of the titin I1 domain. No disulfide linkage was found in ΔAPEG-1. A potential disulfide bond in ΔAPEG-1 would link adjacent strands of the front sheet of the β-sandwich.

To our knowledge, the APEG-1 structure has the highest resolution of all structures of Ig(-like) domains currently present in the Protein Data Bank.

A comparison of the C-terminal loop clusters of ΔAPEG-1 and the I1 domain of titin is of interest. Residues at the interface and the hydrogen bonding network are conserved between the two. The I1 domain of titin forms homodimers in solution and in the crystalline state, but these have a different arrangement from the APEG-1 homodimers and do not appear to be physiologically relevant [28].

The dimerisation of APEG-1 showed a very pronounced salt-dependence, which implies that it is caused by Coulomb interactions. This supports the conclusion that the dimerisation observed with the sedimentation equilibrium technique involves the RGD motif and the salt bridges in the end-to-end contacts of the crystal structure. The dissociation constant of the APEG-1 dimer of 20 μM at physiological ionic strength is quite high and implies that APEG-1 dimerisation could only be transient at physiological conditions.

APEG-1 appears not only as an isolated protein, but also becomes part of the large protein kinase SPEGβ, an alternative product of the APEG-1 gene. The dimerisation of the APEG-1 Ig-like domain could induce antiparallel homodimerisation of SPEGβ. This dimerisation could be stabilized by additional binding sites within the large SPEGβ protein.

The RGD motif is crucial for a number of extracellular protein binding events and cellular adhesion [16-18]. The structural flexibility of C-terminal loops with RGD motifs in published adhesion molecule structures was suggested to allow the molecules to adopt a broad range of conformations in molecular adhesion events [18]. RGD motifs in extracellular proteins have not been described to bind each other. In contrast to the flexibility of extracellular RGD-containing loops, the RGD sequence in APEG-1 forms a defined, rigid 3_10 _helix. Moreover, the APEG-1 RGD motif is only involved in intra-molecular salt bridges, while extracellular RGD motifs have been shown to be involved in inter-molecular salt bridges [32]. Interestingly, the domains Ig14 and Ig17 of twitchin [31] contain the RGD motif, and several domains in the titin I-band contain RGD or KGD motifs [33] at the same position as in APEG-1. APEG-1 is an intracellular protein which points to a possible role of the RGD motif not only in extracellular but also in intracellular protein-protein interactions. However, the way the RGD motif contributes to the homophilic interaction of APEG-1 is obviously quite different from the binding of RGD-containing flexible loops during cell adhesion events.

## Conclusion

Protein engineering facilitated the crystallization of APEG-1. APEG-1 forms a homodimer which is stabilized by salt bridges. This dimerisation is not very strong and its physiogical relevance remains to be established. To our knowledge, the APEG-1 structure has the highest resolution of all structures of Ig(-like) domains currently present in the Protein Data Bank.

## Methods

### Disorder prediction, cloning and expression

Unordered regions were assigned using algorithms available from the DisEMBL™ package [23] and PONDR^® ^[24]. A full-length cDNA fragment and a fragment lacking fourteen residues at the N-terminus of APEG-1 (GenBank:AAH06346) were amplified by PCR from the clone MPMGp800N13557 [34]. Amino acids 15 (Gly) and 16 (Ser) of APEG-1 were not included deliberately into the truncated expression construct, but are encoded by the *Bam*HI restriction site that was used for cloning. For the full-length construct, primers GAA GAT CTA AGC CCA GTC CCA GCC AG and pQE276, sequence GGC AAC CGA GCG TTC TGA AC were used. The truncated construct was created using the primer GAA GAT CTA AGG CAC CCC CCA CCT. The PCR products were cleaved with *Bgl*II and *Not*I and cloned between the *Bam*HI and *Not*I sites of pQTEV (GenBank:AY243506). The resulting plasmids were introduced into *E. coli *SCS1 cells carrying the pRARE plasmid [35]. The full-length construct (2–113, PSF ID 108439) has the ID PSFEp250B082 at the RZPD German Resource Center [36]. The truncated construct of APEG-1 (15–113), termed ΔAPEG-1, was given the PSF ID 111408 and the RZPD ID PSFEp250B117.

### Fermentation and protein purification

The *E. coli *SCS1 clone expressing ΔAPEG-1 was fermenter-grown to an OD_600 _of 8 in 4 l of SB medium (12 g/l bacto-tryptone, 24 g/l yeast extract, 0.4% (v/v) glycerol, 17 mM KH_2_PO_4_, 72 mM K_2_HPO_4_) supplemented with 20 μg/ml thiamine, 100 μg/ml ampicillin and 34 μg/ml chloramphenicol. Protein expression was induced with 1 mM isopropyl-β-D-thiogalactopyranoside (IPTG) for 3 h at 37°C. Cells were pelleted by centrifugation and washed in extraction buffer (20 mM Tris-HCl, pH8.0, 300 mM NaCl, 0.5 mM EDTA (ethylenediaminetetraacetic acid), 1 mM PMSF (phenylmethylsulfonylfluoride), 5 mM 2-mercaptoethanol). The cells were lysed, and cell lysates and proteins were stored at 4°C. Protein purification steps were performed at room temperature. The pellets of the protein-expressing cells were resuspended in a 4 to 6-fold volume of extraction buffer. Lysozyme was added to 0.4 mg/ml and cells were disrupted by sonification. Cellular debris was removed by centrifugation (55,000 × *g*, 45 min) and the supernatant was filtrated through cellulose nitrate (0.45 μm). The pH of the solution was adjusted to pH7.4 and the extract was applied to a 10-ml TALON Superflow 16/20 column (BD Biosciences) equilibrated with buffer (20 mM Tris-HCl, pH 7.4, 500 mM NaCl, 10 mM imidazole). The protein was eluted using buffer containing 50 mM NaCl, 200 mM imidazole, 0.5 mM EDTA, and 1 mM DTT (dithiothreitol). TEV protease (1:40) was added to effect removal of the His_6 _tag (overnight, 4°C). The protein solution was diluted 5-fold in 20 mM Tris-HCl, pH7.4 and applied to a 4 ml-POROS 20 HQ anion-exchange chromatography column (Applied Biosystems). The flow-through of the anion-exchange chromatography was applied to a POROS 20 S cation-exchange chromatography column (8 ml volume), and ΔAPEG-1 was again found in the flow-through. After size-exclusion chromatography (Superdex75 XK 16/60, Amersham) the protein yield was 23.5 mg. Samples were stored in 15 mM Tris-HCl, pH7.4, 50 mM NaCl, 0.1 mM EDTA, 2 mM DTT, 0.02% NaN_3_.

### Biophysical experiments

Monodispersity of the sample was confirmed by dynamic light scattering experiments (spectroscatter 201, RiNA RNA-Network GmbH, Berlin, Germany). Thermal stability and the midpoint of the unfolding transition were determined by differential scanning calorimetry (capDSC, MicroCal, LLC).

Quaternary structure analyses were done with the sedimentation equilibrium technique using an analytical ultracentrifuge XL-A (Beckman, Palo Alto CA) as described earlier [37,38]. About 70 μL APEG-1 or ΔAPEG-1 protein dissolved in (15 mM Tris-HCl, pH 7.4, 0.1 M NaCl, 0.1 mM EDTA, 0.2% NaN_3_) were centrifuged 2 h in 6-channel cells at 32,000 rpm (overspeed) followed by 26–30 h equilibrium speed at 28,000 rpm and 10°C. The radial absorbance distributions at sedimentation equilibrium were recorded at three different wavelengths between 270 and 300 nm and fitted globally to the molecular mass using our program POLYMOLE [37]. In case of a monomer-dimer equilibrium the molecular mass values can be considered as weight average data *M*_w _= (*c*_m_· *M*_m _+ *c*_d_·*M*_d_)/(*c*_m_+ *c*_d_) defined by the molecular masses of monomers and dimers and their partial concentrations from which the equilibrium dissociation constant *K*_d _was determined.

### Crystallization and data collection

Crystallization trials using the vapor diffusion method were set up by a semi-automated dispensing system [39] in a 96-well Greiner Crystal Quick™ low-profile plate. Crystals were obtained from droplets comprising 400 nl of protein (57.2 mg/ml) plus 400 nl of (30% polyethylene glycol monomethylether 2000, 200 mM (NH_4_)_2_SO_4_, 100 mM Na-acetate, pH 4.6) equilibrated against 75 μl of reservoir solution. Crystals grew in multiple fan-like clusters of thick plates within 7 days at 20°C. The crystals belong to the monoclinic space group C2 with unit cell dimensions of *a *= 81.5 Å, *b *= 25.5 Å, *c *= 42.5 Å and *β *= 104.6°. One molecule in the asymmetric unit corresponds to a *V*_M _value [40] of 1.8 Å^3^/Da assuming one molecule in the asymmetric unit and a solvent content of 32%. A portion of the crystal was extracted from the cluster and was briefly transferred to a cryo-protectant solution consisting of the mother liquor supplemented with 10% PEG 400. The crystal was flash-cooled in a liquid nitrogen stream at 100 K and the beam was centered to one edge of the crystal to obtain diffraction from a single crystal. Diffraction data were collected on a MAR345 imaging plate detector using the synchrotron source at a wavelength of 0.9184 Å (beamline PSF-ID14.2 at BESSY, Free University, Berlin). A dataset to 0.96-Å resolution was obtained in two sweeps in order to optimize the high-resolution intensities and to obtain complete low-resolution data. The measured data were integrated, scaled and merged using the programs DENZO and SCALEPACK [41] (Table 1).

### Molecular replacement, model building and refinement

The crystal structure of ΔAPEG-1 was determined by molecular replacement using the program Auto-AMoRe [42] implemented *via *the CCP4 GUI suite [43]. A homology model was constructed using SWISS-MODEL in the program's default settings [44] based on PDB coordinate sets 1FHG, 1BIH and 1CS6. The derived model was used as the starting model for molecular replacement phasing with diffraction data in the resolution range 8.0-3.0 Å. A clear solution for the single molecule in the asymmetric unit was obtained as indicated by the correlation coefficient of 0.446 for the best solution and 0.317 for the second best solution. Density improvement and removal of model bias along with automatic model tracing was performed by the free-atom refinement method in ARP/wARP [45] using data to 1.7 Å resolution. The electron density map allowed 97% of the model to be built automatically. The model was subsequently completed manually using O [46] and was refined with isotropic temperature factors to atomic resolution (0.96 Å) using REFMAC [47]. Alternative side-chain conformations were assigned for residues S25, S40, L54, Q58, R102, E109, and water molecules were added into positive difference density if they were hydrogen-bonded to polar atoms. During the final stages of the refinement, anisotropic temperature factors were applied to the non-hydrogen atoms and hydrogens were included as riding atoms. The final refinement statistics are shown in Table 1. The relatively high *R *factors are associated to the weak and incomplete diffraction data at high resolution. The final model contains 96 residues and 151 water molecules. No electron density was visible for the terminal residues G15, S16 and E113, and they were excluded from the model. The stereochemical quality of the model was assessed using the programs PROCHECK [48] and SFCHECK [49]. The atomic coordinates for the final model and experimental structure factors are accessible under the PDB code 1U2H. Figures were prepared using Molscript [50] and Pymol [51].

### Database search for high resolution immunoglobulin structures

We have searched the PDB and SCOP databases for high resolution structures of immunoglobulin domains. A list of structures with at most 1.1 Å resolution and at least 50 amino acids length was compared to a list of structures of the immunoglobulin superfamily obtained from SCOP 1.69 [52].

## Authors' contributions

VS made the original clone. CS preformed prediction of the disordered region and designed the deletion mutant. FG prepared the proteins, FHN performed light scattering and calorimetry experiments. YR crystallized the protein. BAM solved the structure and prepared the initial draft of the manuscript. UH and KB conceived of the study, and participated in its design and coordination. KB prepared the final manuscript. JB measured *K*_d _values by ultracentrifugation.

## Figures and Tables

**Table 1:** Data Collection and Refinement Statistics

Wavelength (Å)	0.9184
Resolution range (Å)	20.0 0.96
Number of measured reflections^¶^	183,130 (3,132)
Unique observations^¶^	46,733 (1,651)
Redundancy^¶^	3.9 (1.9)
Completeness (%)^¶^	89.7 (47.3)
*R*_merge_^¶^	0.07 (0.16)
Average *I*/σ(*I*)^¶^	29.9 (4.5)
	
Number of reflections for refinement	43,956
Number of reflections in *R*_free _set	2,384
*R *factor ^†^	0.160
Free *R *factor ^‡^	0.172
Overall correlation coefficient	0.957
Free correlation coefficient	0.953
r.m.s.d. bond lengths (Å)	0.015
r.m.s.d. bond angles (°)	1.78
Number of non-hydrogen atoms	765
Average *B *factor (Å^2^)	7.3

^¶^Statistics for the highest resolution bin (0.98 Å – 0.96 Å) are given in parentheses.

^†^*R *= Σ Σ_h_||*F*_o_(h)| - *k*|*F*_c_(h)||/Σ_h_|*F*_o_(h)|

^‡^Free *R *factor was calculated using a 5% randomly selected subset of the total number of reflections.
